# Reconfiguring Abortion Infrastructures: An STS Analysis of Trends in Medical and Surgical Abortion in Post‐Legalisation Contexts

**DOI:** 10.1111/1467-9566.70288

**Published:** 2026-10-27

**Authors:** Aideen O'Shaughnessy

**Affiliations:** ^1^ School of Law, Politics, & Society University of Lincoln Lincoln UK

## Abstract

The growing dominance of medical abortion in liberal and post‐legalisation contexts raises important questions about patient choice, reproductive autonomy and the organisation of abortion care. Whilst existing scholarship has examined the sociotechnical implications of medical abortion, contemporary shifts in relation to surgical abortion remain comparatively understudied. This research note proposes the notion of ‘abortion infrastructures’ as a sensitising concept for examining how abortion technologies are embedded in, and recursively reconfigure, broader sociotechnical systems of care. Drawing on feminist science and technology studies (STS), it develops four interconnected analytical dimensions: the relational embeddedness of abortion methods within medico‐legal systems; the emergence of new spatial, temporal and institutional norms of provision; the relational differentiation of medical and surgical abortion; and the classificatory work through which these methods acquire distinct meanings and institutional positions. This framework directs attention to how resource constraints, clinical protocols, professional practices and moral assumptions may privilege medical abortion whilst rendering surgical abortion increasingly inaccessible. It also problematises understandings of method ‘choice’ which overlook the infrastructural conditions shaping available options. By foregrounding the normative and relational dimensions of abortion provision, this note advances a feminist infrastructural approach to trends in abortion methods and identifies directions for future empirical research.

## 1 Introduction: The Revolution in Abortion Methods

Amid political campaigns to curtail reproductive freedom and the rise of transnational feminist movements for self‐managed abortion, legal frameworks, clinical practices and institutional priorities in abortion have undergone significant transformation. In this context, feminist scholars have continued to attend to technoscientific innovation as a key site mediating shifts in reproductive rights. The past two decades have witnessed a revolution in abortion methods with medical abortion (MA) largely ‘replacing’ surgical abortion in several contexts (Footman 2023; Popinchalk and Sedgh 2019). Medical abortion involves the termination of pregnancy using ‘abortion pills’—mifepristone and misoprostol—and typically requires the patient to ‘self‐manage’ the process at home. Surgical abortion is a clinical procedure, involving sedation or general anaesthetic, which entails removing the pregnancy primarily using suction techniques.

Developed originally as a progesterone receptor antagonist, mifepristone was first approved for use in abortion in France in 1988 (Magon et al. 2012). At the same time, Brazilian feminist activists adapted misoprostol (originally marketed as a stomach ulcer drug) as an abortifacient (Assis and Larrea 2020). Following contentious regulatory debate, the US Food and Drug Administration approved mifepristone for use alongside a prostaglandin (later standardised as misoprostol) for abortion in 2000 (Calkin 2023). The development of medical abortion is widely understood to have reshaped control over reproductive decision‐making and facilitated greater access to abortion, particularly in low‐resource and legally restrictive settings (Assis and Larrea 2020).

Less examined is the turn towards medical abortion in liberal and post‐legalisation contexts. In England and Wales, the percentage of medical abortions increased from 49% in 2013 to 87% in 2023, whilst the proportion of surgical abortions fell dramatically from 51% to 13% over this period (Department of Health and Social Care 2026). In Ireland, a government report stated that ‘nearly all’ cases of early termination of pregnancy performed since legalisation in 2018 were medical abortions, and that medical management is also standard practice for terminations beyond 12 weeks (O’Shea 2023). In health policy research, this growing reliance on medical abortion is attributed to the removal of legal restrictions on medical abortion (including the expansion of telemedical abortion during COVID‐19) and to resource constraints, including limited opportunities for surgical abortion training, reduced theatre availability and cost‐driven commissioning practices (Footman 2024; O’Shea 2023).

Existing research on trends in abortion methods tends to treat medical and surgical abortion as discrete sets of material objects, rather than as technologies which are co‐produced within, and which themselves recursively reconfigure the broader landscape of care. This research note calls for greater attention to the relationality and embeddedness of abortion methods within broader sociotechnical systems. It proposes the notion of *abortion infrastructures* as a sensitising concept to examine the relational, ecological and sociotechnical webs of abortion care, comprising spatial arrangements, patients, doctors, technologies and clinical protocols, amongst other objects and relations. It suggests that this ‘infrastructural’ lens may be useful to examine the association between a growing reliance on medical abortion in post‐legalisation landscapes and shifts in the spatio‐temporal, institutional and moral norms of abortion provision, and how these shifts may reposition surgical methods of pregnancy termination in contemporary models of care.

## 2 Reproductive Autonomy and Navigating ‘Choice’: Patient‐Centred Perspectives

The WHO in its 2022 Abortion Care Guidelines outlined ‘choice’ in relation to abortion method as a key component of quality care (World Health Organization 2022). In relation to surgical abortion, the WHO recommends either electronic or manual vacuum aspiration for pregnancies under 14 weeks and dilation and evacuation (D&E) for pregnancies greater than 14 weeks gestation. Manual vacuum aspiration (MVA) uses a handheld syringe to create suction for uterine evacuation and can be carried out in an ambulatory setting; whilst electronic vacuum aspiration, which is performed in a theatre space, uses a machine‐powered pump. Whilst medical abortion is safe and effective at various gestational ages, surgical provision may be required where contraindications exist or when complications from MA arise.

Medical and surgical abortion offer markedly different experiences in terms of the duration of the process, pain, and bleeding. Research suggests patient preferences may vary according to age, socio‐cultural background, and previous abortion experiences, and that greater involvement in decision‐making around method may improve patient wellbeing (Footman 2024). In contexts with high ratios of medical abortion then, questions about patient ‘choice’ in method must be raised (Footman 2023). Emerging evidence in contexts including Ireland suggests that abortion‐seekers have felt ‘pressured’ into accepting medical abortion when surgical abortion was their preferred treatment (Grimes and Abortion Rights Campaign 2021). In Northern Ireland, where surgical abortion is ‘largely unavailable’, surgical abortion‐seekers are required to travel to England to access care (Amnesty International UK 2023).

The question of the differential stigmatisation of medical and surgical abortion methods also requires further analysis. Currently, there is insufficient data on a global scale to argue that structural stigma effects method availability (Footman et al. 2024). Emerging evidence from contexts including Ireland and the US, however, demonstrates that medics providing surgical abortion experience higher levels of stigma in hospital environments, and that practitioners find the provision of medical abortion ‘emotionally easier’ (Dempsey et al. 2021; McCaffrey 2024; Greenberg and Nothnagle 2018). A lack of ‘willingness’ amongst the obstetrician community in Ireland to perform surgical abortion was also highlighted as a barrier to choice of method in a recent government report (O’Shea 2023).

## 3 Revisting ‘Scripts’ and ‘Interpretive Flexibility’: New Directions in Feminist Analyses

Feminist STS research has heavily privileged medical abortion, leaving contemporary trends in surgical abortion comparatively understudied. Existing scholarship draws heavily on Akrich's (1992) concept of ‘inscription’—which defines the instructions, expectations, and assumptions designers inscribe into technical artefacts about how they should be used, by whom, and in what context. As Calkin (2023) argues, the abortion pill ‘exists at the centre of multiple “scripts” which convey different ideas about the device, its users and its users' bodies’ (10). Attending to its ‘interpretive flexibility’ illuminates how the meanings ascribed to the ‘abortion pill’ are contingent on a specific ‘constellation of actors’, practices, and environments in which medication abortion circulates—such that the same technology can be framed as a ‘death pill’ by anti‐abortion activists in the United States, even as it is simultaneously heralded within Mexican biomedicine as the ‘gold standard’ of care (Pinch and Bijker 1984; Berro Pizzarossa and Nandagiri 2021; Belfrage 2023).

In their study on the changing ‘body work’ of abortion published in this journal, Purcell et al. (2017) explore how—as medical abortion is progressively appropriated within neoliberal healthcare systems—abortion‐seekers are increasingly *responsibilised* as ‘self‐managing’ reproductive subjects. Offering a comparative analysis of medical and surgical abortion, the authors argue that medical abortion protocols shunt the ‘body work’ of abortion ‘down the medical hierarchy’ and are emblematic of a ‘dematerialising tendency’ in contemporary provision with patients expected to monitor, assess, and manage their abortions ‘at home’ (Purcell et al. 2017, 80). That medical abortion allows the delegation of the ‘hands‐on’ work of abortion to women themselves is theorised as one factor underlying a growing institutional preference for this method (Purcell et al. 2017; McCaffrey 2024).

This restructuring of the ‘workflow’ of abortion through medical management may alter patient and practitioner experiences in ways that have implications for how abortion stigma is produced and negotiated in practice, although these dynamics require further empirical investigation. I suggest that this reconfiguration of the division of labour of abortion may be understood as one indication of how shifting trends in abortion methods facilitate a broader rewiring of *abortion infrastructures*, mediating the emergence of new *modalities* of abortion care. In historically liberal and post‐legalisation landscapes, these modalities represent novel enactments of medical and surgical abortion which require further study to understand their effects on the medical, legal, and socio‐cultural reframing of abortion and reproductive autonomy.

## 4 Towards an Infrastructural Analysis of Trends in Abortion Methods

This research note offers a preliminary STS‐informed framework for thinking about contemporary trends in abortion methods, organised around four interconnected analytical dimensions which may provide a productive starting point for further research. First, mobilising Star's (1999) definition of infrastructure as a ‘fundamentally relational concept, becoming real in relation to organised practices’, I propose conceptualising medical and surgical abortion technologies as nodes within broader *abortion infrastructures*. (380). Conceptualising medico‐legal systems of abortion provision as ‘infrastructure’ allows us to better trace the *co‐production* of medical and abortion technologies within these contexts and to understand how these technologies become distinctively usable and intelligible across diverse settings.

Second, I suggest that in post‐legalisation and liberal abortion regimes, medical abortion models constitute a novel *enactment* of medical abortion technologies which potentially reshapes the spatio‐temporal, institutional, and social norms about what an abortion is, how/where it should be carried out, and what it should look/feel like (Mol 2002). Purcell et al.’s (2017) argument that increasing medical abortion use represents a ‘dematerialising tendency’ in abortion provision where patients are expected to manage their abortions *beyond* the clinic, exemplifies these new spatio‐temporal arrangements. Growing institutional reliance on medical abortion then may reinforce assumptions that abortion should occur ‘early’, ‘at home’ and be minimally demanding of the time and resources of OBGYNs.

Third, I propose that the integration of new abortion technologies *recursively reconfigures* broader abortion infrastructures. Concretely, as medical abortion is integrated in historically liberal or post‐legalisation landscapes, this technology changes the infrastructural conditions in which preexisting termination of pregnancy methods (specifically, surgical models of abortion) are encountered. As medical abortion modalities are increasingly framed in public health, and policy discourse as ‘cheaper’, ‘more private’, as well as emotionally ‘easier’ for healthcare practitioners, surgical abortion is positioned as ‘burdensome’, ‘uncomfortable’, and technically more ‘complex’ (Purcell et al. 2017; Greenberg and Nothnagle 2018). These developments may be understood as a process of relational differentiation or inscription which is not produced by either technology in isolation, but which emerges dynamically within the broader abortion infrastructure.

Finally, reflecting on the role of *classificatory systems* in shaping abortion infrastructures, I want to call attention to the institutional work that the categorisation of ‘medical’ and ‘surgical’ abortion performs. As this distinction travels through healthcare systems, it becomes attached to particular kinds of staff, spaces, affects and ideas about risk, labour, and time. These classifications become tools which serve socio‐legal functions (Bowker and Star 2000). Even the categorisation of MVA as ‘surgical’, for example, means that despite not requiring either a theatre or general anaesthetic, its provision remains highly professionalised and policed. We must ask then, what does it mean to offer abortion‐seekers a ‘choice’ of method when surgical abortion is rendered practically inaccessible through skills shortages, commissioning practices, and resource constraints? This classification system combined with a lack of real infrastructural choice becomes self‐reinforcing as the lack of surgical capacity itself becomes evidence of its undesirability.

## 5 Conclusion: On the Normativity of Abortion Infrastructures

As Star (1999) argues, infrastructures are political and normative; they shape what sorts of practices and relations become possible or expected. In the context of a growing reliance on medical abortion in liberal and post‐legalisation contexts, it is worth considering how these new modalities of abortion will reconfigure the broader sociotechnical landscape. Such developments may involve not only the redistribution of power, labour and agency, but the moral and affective reclassification of various forms of pregnancy termination. This note then calls for further empirical analyses of these trends which go beyond critiques of medicalisation and questions of stratified access to consider how certain types of abortion become preferred or quietly imposed, and to determine the profound implications of these trends for reproductive rights.

## Author Contributions

**Aideen O'Shaughnessy:** conceptualization, investigation, writing – original draft, methodology, writing – review and editing.

## Funding

The author has nothing to report.

## Ethics Statement

The author has nothing to report.

## Conflicts of Interest

The author declares no conflicts of interest.

## Data Availability

The author has nothing to report.
